# Identifying research areas in peripheral artery disease treatment—a Delphi consensus approach

**DOI:** 10.1186/s42155-026-00762-z

**Published:** 2026-08-27

**Authors:** Stefan Müller-Hülsbeck, Franziska Reiter, Andreas H. Mahnken, Nathalie Kaufmann, Robert A. Morgan

**Affiliations:** 1 Institut Für Diagnostische Und Interventionelle Radiologie Und Neuroradiologie, Malteser Fördeklinikum St. Katharina, Flensburg, Germany; 2 Next Research, Vienna, Austria; 3 Diagnostic and Interventional Radiology and Nuclear Medicine, St. Josef-Hospital, Klinikum Der Ruhr-Universität Bochum, Bochum, Germany; 4 Department of Vascular and Interventional Radiology, St George’s University Hospitals NHS Foundation Trust, Blackshaw Road, London, SW17 0QT UK

**Keywords:** Delphi panel, PAD, Atherectomy, Consensus statements, Endovascular therapy

## Abstract

**Background:**

The multitude of existing techniques for the endovascular treatment of peripheral artery disease (PAD), as well as newly emerging technologies combined with the diversity of patient profiles highlight the need for further research in this area. We conducted a Delphi consensus study, an established method utilising an iterative approach to reach consensus within a group of experts over several rounds, to identify gaps and new areas of research in the field of PAD.

**Results:**

Using a four-round Delphi process, we started with 17 statements proposed by an expert panel to be rated on a 5-point Likert scale by EBIR-ES holders. We defined two consensus metrics to measure the agreement within the group: as > 75% concordance or > 60% certainty. Six statements that did not meet this threshold after round 1 underwent further rounds of expert feedback, discussion, and reassessment.

Fifty-two EBIR-ES holders evaluated the initial statements and 36 contributed to the final consensus. While certainty was stable across all rounds, an increase in concordance was seen in 4 (67%) statements. As a supplement to the traditional Delphi method, we interpreted the decrease in concordance for 2 (33%) statements as areas in need of further research.

**Conclusions:**

The Delphi consensus method is an effective method for discovering potential research topics. Through iterative feedback, it encourages experts to refine their views and promotes a productive exchange of ideas. We identified the use of filter wires for atherectomy, as well as the comparison between atherectomy devices as key areas needing further research. Moreover, these findings underscore the value of developing formalised, multidisciplinary consensus guidelines to guide clinical practice.

**Supplementary Information:**

The online version contains supplementary material available at https://doi.org/10.1186/s42155-026-00762-z.

## Background

An estimated 130 million people currently suffer from peripheral artery disease (PAD), with the numbers projected to triple in the next 25 years [1, 2]. The burden of PAD in each country puts an enormous weight on health systems in terms of the resources required to treat and prevent chronic limb-threatening ischaemia (CLTI) and lower limb amputation [3, 4].

Interventional radiologists play a major role in the treatment of PAD along with colleagues in vascular surgery and angiology [5]. Endovascular specialists must master numerous techniques and technologies to be able to effectively treat the whole range of arterial pathologies that make up the spectrum of CLTI [6–8].

Cutting-edge research is vital for the development and optimal provision of endovascular therapies for PAD. Highlighting key gaps in the evidence landscape is essential to select the most fertile areas for research to optimally benefit patients. Delphi methodology is an established research tool to reach consensus among a panel of experts on a complex topic [9]. Delphi methodology involves anonymous surveys, controlled feedback on group responses and multiple rounds of questionnaires until a consensus is reached or the level of agreement is deemed sufficient [10].

The multitude of existing techniques for the treatment of PAD, as well as newly emerging technologies make it challenging to select the most deserving topics for research in PAD. We conducted a Delphi consensus study to identify gaps and required new areas of research in the field of PAD. The aim of this article is to describe the Delphi process that we used and the outcomes of our project.

## Materials and methods

### Delphi process

An expert group of six interventional radiologists was tasked with proposing the initial statements to be the subject of a Delphi consensus study. Seventeen initial statements (Table 1) were agreed upon. These were categorised into five topic segments: (A) Atherectomy and lesion preparation, (B) Atherectomy Techniques and Devices, (C) Specific Arterial Segments and Treatments, (D) PAD Management, and (E) Clinical Practice Operations.

**Table 1:** List of the initial 17 statements and results from round 1

	**Statement**	**Certainty**	**Concordance**	**Result**	**Selected**
1	Lesion preparation is necessary before DCB angioplasty in calcified lesions	88%	91%	Agree	
2	Lesion preparation should be considered for uncalcified lesions before DCB angioplasty	85%	75%	Agree	
3	There is a clearly defined ideal patient profile for atherectomy procedures	81%	62%	Disagree	Yes
4	The use of filter wires during atherectomy procedures is essential to prevent complications	75%	69%	Agree	Yes
5	Certain lesion types should not be treated with atherectomy	88%	96%	Agree	
6	The choice of atherectomy devices significantly impacts patient outcomes	60%	77%	Agree	Yes
7	IVL is a comparable alternative to atherectomy for specific patient scenarios	60%	84%	Agree	Yes
8	Using a filter wire during common femoral artery atherectomy is necessary	71%	69%	Agree	
9	The selection of stents for the popliteal segment should be based on specific criteria	100%	100%	Agree	
10	In-stent restenosis should be treated aggressively to prevent further complications	94%	86%	Agree	
11	There are clear indications for when to treat occluded stents versus opting for bypass surgery	81%	71%	Disagree	Yes
12	A specific number of vessels should be revascularised Below-the-Knee (BTK) for optimal patient outcomes	88%	50%	Agree/ Disagree	Yes
13	There are established strategies for managing a "desert foot" that should be universally applied	75%	69%	Disagree	
14	Having an outpatient clinic is essential for the optimal management of interventional radiology patients	87%	91%	Agree	
15	Regular ward rounds are crucial for maintaining high-quality patient care in interventional radiology	87%	100%	Agree	
16	Ambulatory treatments and aftercare are vital components of the patient care pathway in interventional radiology	94%	100%	Agree	
17	A systematic follow-up process is crucial for the long-term success of interventional radiology treatments	98%	98%	Agree	

#### Panel selection

To select panel members with high expertise in the field of endovascular IR techniques, invitations to participate in the Delphi consensus panel were sent to all holders of the EBIR-ES certificate (European Board of Interventional Radiology–Endovascular Specialist). In addition, the members of the Endovascular Subcommittee of CIRSE were invited to participate. Fifty-two out of the 71 invited physicians accepted the invitation. The final panel consisted of interventional radiologists from 19 different countries (4 non-European countries). A list of panel members can be found in Supplementary Table S1.

#### Delphi rounds

Prior to the start of the Delphi process, an expert group comprising eight senior interventional radiologists developed a set of 17 statements for evaluation by the consensus panel. Candidate topics were identified during two structured brainstorming rounds, with the aim of addressing areas of ongoing controversy in the management of peripheral arterial disease. The final set of statements was approved by consensus among all members of the expert group. No additional predefined or formalised criteria were used for statement selection.

In the first round, 52 panel members rated all 17 statements on a 5-point Likert scale (Strongly Disagree, Disagree, Neutral, Agree, Strongly Agree). Consensus scores were defined based on two measurements:

1. Certainty—defined as the percentage of answers that are not “Neutral”
2. Concordance—defined as the percentage of non-neutral answers that lead to a result (i.e. most members selected “strongly agree”/“agree” or most members selected “strongly disagree”/”disagree”).

Based on these criteria, six statements with a low consensus score (certainty ≤ 60% or concordance ≤ 75%) were selected for subsequent rounds [9, 11].

In round 2, all panel members were asked to give a background explanation for their opinions on each of the six selected statements. All answers were reviewed manually, and an anonymised summary of the given opinions together with a list of the mentioned points was sent to all panel members in preparation for the discussion round (round 3).

The discussion round was held in an online setting with 13 panel members participating. Each statement was first introduced with the results from rounds 1 (distribution on Likert scale ratings) and round 2 (summary of the stated opinions) before participants were invited to share their opinions. No formal review of the literature was conducted as preparation for the discussion. The discussion was moderated in a way that ensured that participants had equal opportunity to share their opinions. During discussion the ambiguity of statements 3 and 12 was noted the participants. While interpretation of these statements was discussed and clarified, no adaptation of the wording was implemented. A summary of the discussion including the main arguments for each position was sent to all panel members after its conclusion.

In round 4, panel members were given the opportunity to reassess their opinions on the selected six statements (5-point Likert scale from Strongly Disagree to Strongly Agree). Individualised forms were shared, detailing the panellist’s previous Likert scale ratings from round 1 and providing the option for new ratings. Panel members who changed their ratings were also encouraged to share the reasons for this change of mind. We defined “positive change” as a change in Likert scale rating towards “Strongly Agree” and “negative change” as a change in Likert scale rating towards “Strongly Disagree”.

#### Participation and drop-out

**Fig. 1:**
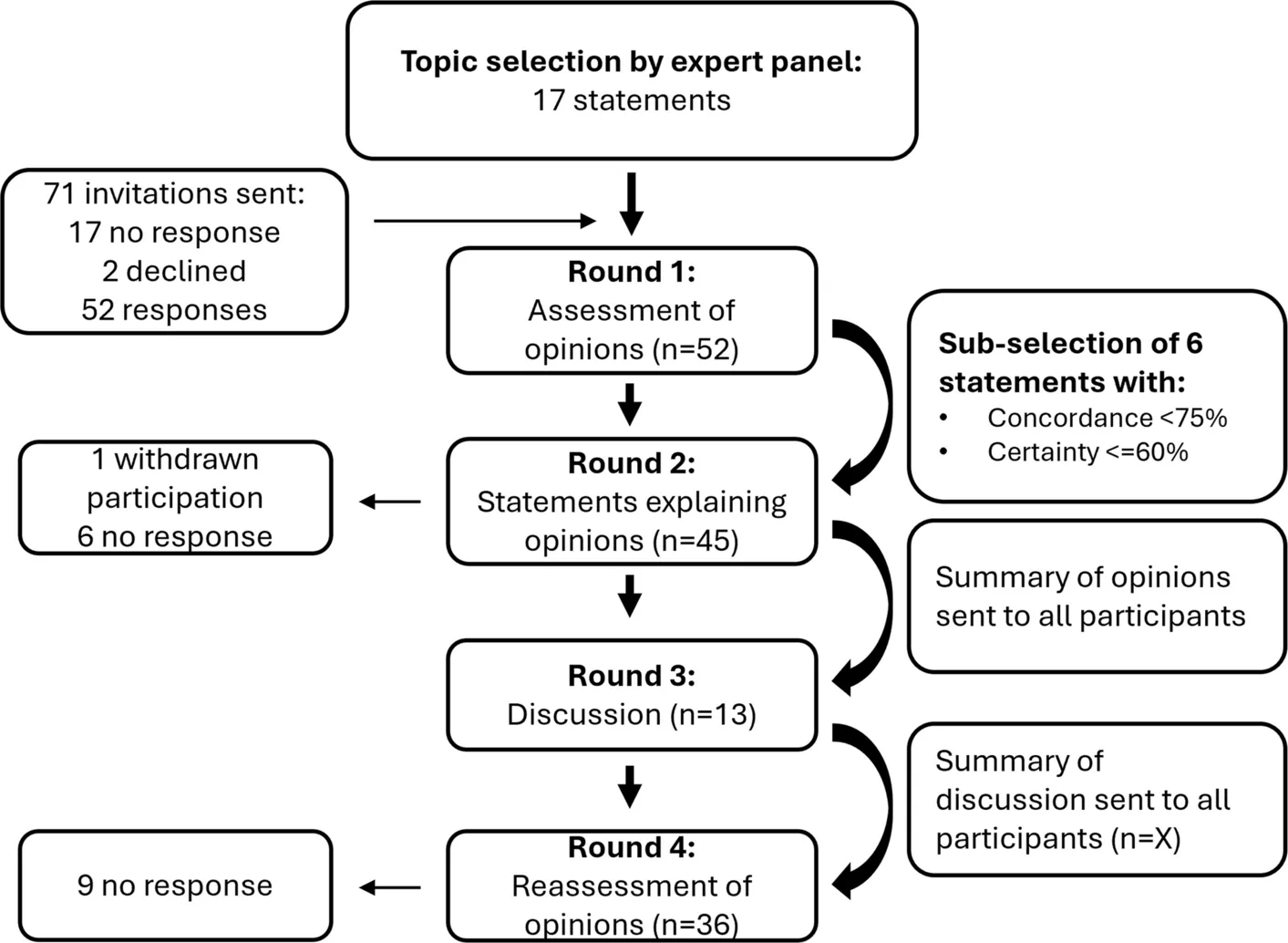
Flowchart of the four-round Delphi process. n denotes the number of participants in each round

To keep participation in the Delphi process at an acceptable level, regular reminders were sent to participants. Fifty-two physicians agreed to participate in the Delphi process and rated the initial 17 statements (Fig. 1). Participation dropped to 45 in round 2. Panel members who did not submit answers for round 2 were disqualified from participating further. The discussion round (round 3), which was held in an online format, was attended by 13 physicians. A summary of the discussion results was sent to the remaining 45 participants. A further 9 dropped out in round 4, leaving 36 panellists who completed all rounds.

### Data collection

The survey platform Alchemer (Alchemer: Enterprise Online Survey Software & Tools, Louisville, US, CO 80027; https://www.alchemer.com/) was used to collect data in round 1. For all subsequent rounds, forms were prepared for each participant and shared with them to complete via an online platform. All submitted answers were reviewed and summarised manually. Summaries were sent out to all panel members via email.

### Analysis

All answers collected in Alchemer were exported from the platform in one master table. Answers collected in subsequent rounds were manually transcribed into an Excel table. All later analysis steps were performed using R version 4.3.2. Data are presented as counts and percentages of responses for tables and figures. Data were analysed and plotted using R Studio under version 2025.5.1.513 (*Posit Software, PBC, Boston, MA. URL *http://www.posit.co/).

Opinions and statements from rounds 2 and 4 as well as the discussion round were manually summarised by the authors.

### Data availability

The datasets supporting the conclusions of this article are included within the article (and its additional files).

## Results

### Round 1

#### Initial assessment

**Fig. 2:**
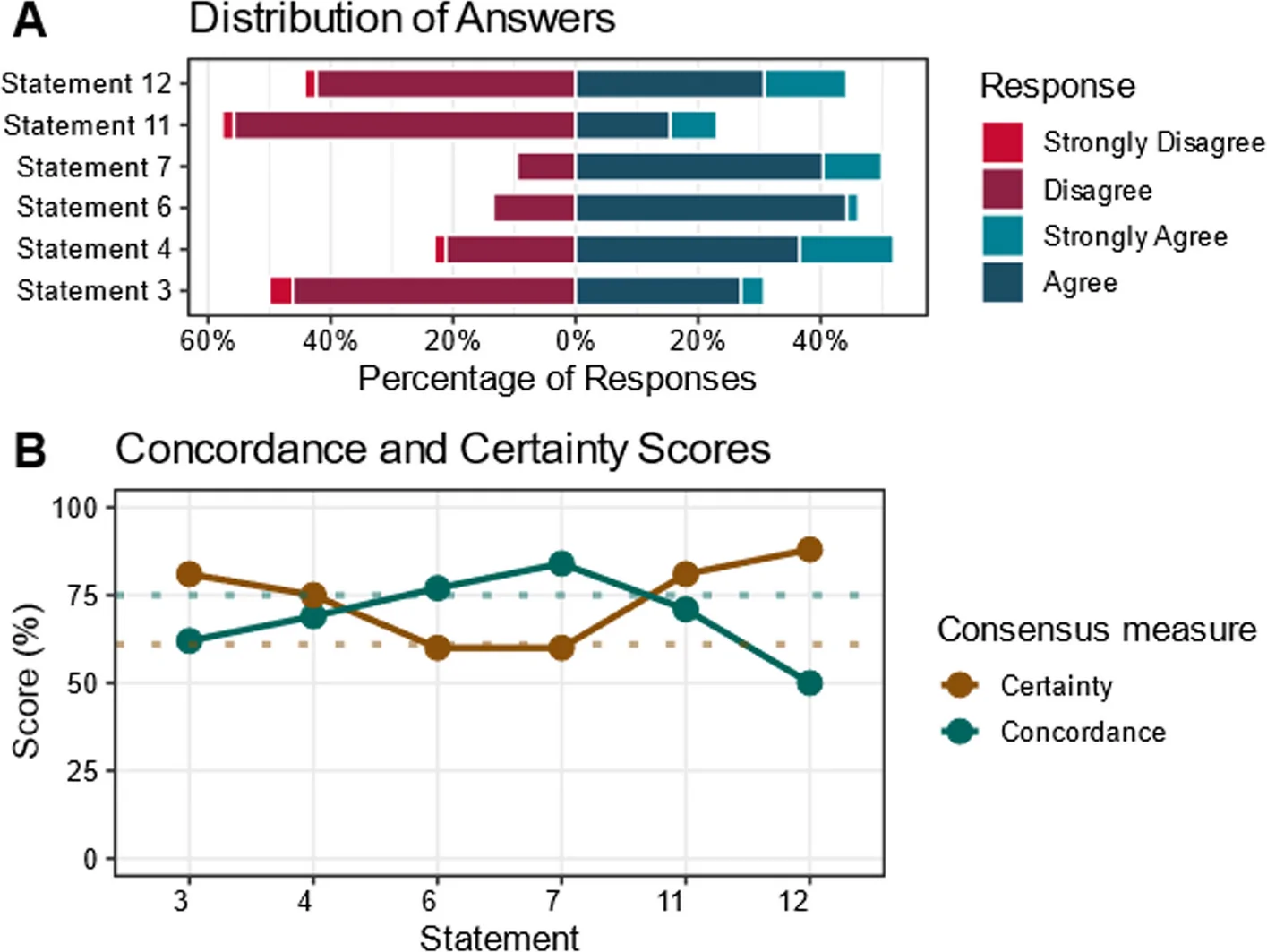
Consensus scores of the six selected statements in round 1. **A** shows the distribution of Likert scale ratings (excluding neutral answers). **B** shows the certainty (brown) and concordance (teal) score for each statement. Cut-offs that were applied for selection (≤ 75% concordance or < 60% certainty) are shown as dashed lines

Five of the initial 17 statements had a concordance score of > 95% (Statements 5, 9, 15, 16, 17), including three that reached 100% agreement (Table 1). As the Delphi panel aimed to identify gaps in PAD evidence, six statements with the lowest consensus scores (certainty ≤ 60% or concordance ≤ 75%) were selected for subsequent rounds (Fig. 2). Statement 13 (“There are established strategies for managing a ‘desert foot’ that should be universally applied.”) was excluded from follow-up. The topic was deemed to be too complex to be included with the other statements. However, the subject was highlighted as important and should be addressed in a future project. To avoid redundancy with statement 4, statement 8 (“Using a filter wire during common femoral artery atherectomy is necessary.”) was also excluded from follow-up rounds. The distribution of Likert scale ratings (excluding neutrals) is shown in Fig. 2A. Four statements [3, 4, 11, 12] had a concordance score < 75% while two [6, 7] were included due to their low certainty score (≤ 60%) (Fig. 2B). Of the selected statements, four addressed atherectomy and intravascular lithotripsy:

- 3: “There is a clearly defined ideal patient profile for atherectomy procedures.”
- 4: “The use of filter wires during atherectomy procedures is essential to prevent complications.”
- 6: “The choice of atherectomy devices significantly impacts patient outcomes.”
- 7: “IVL is a comparable alternative to atherectomy for specific patient scenarios.”

and two focused on PAD management:

- 11: “There are clear indications for when to treat occluded stents versus opting for bypass surgery.”
- 12: “A specific number of vessels should be revascularised Below-the-Knee (BTK) for optimal patient outcomes.”

### Round 2

#### Opinions

In *round 2*, the response rate was 86%. The submitted answers were reviewed and summarised (Supplementary Table S2). Panel members cited the lack of high-quality studies and clinical guidelines in four of the six statements [3, 6, 7, 11] as a reason for rating them at “Disagree” or “Strongly Disagree”. Similarly, panel members who selected “Neutral” for these statements also attributed their rating to the lack of high-quality evidence. Experts who did not submit their answers within the given timeframe were excluded from subsequent Delphi rounds.

### Round 4

#### Discussion and reassessment

**Fig. 3:**
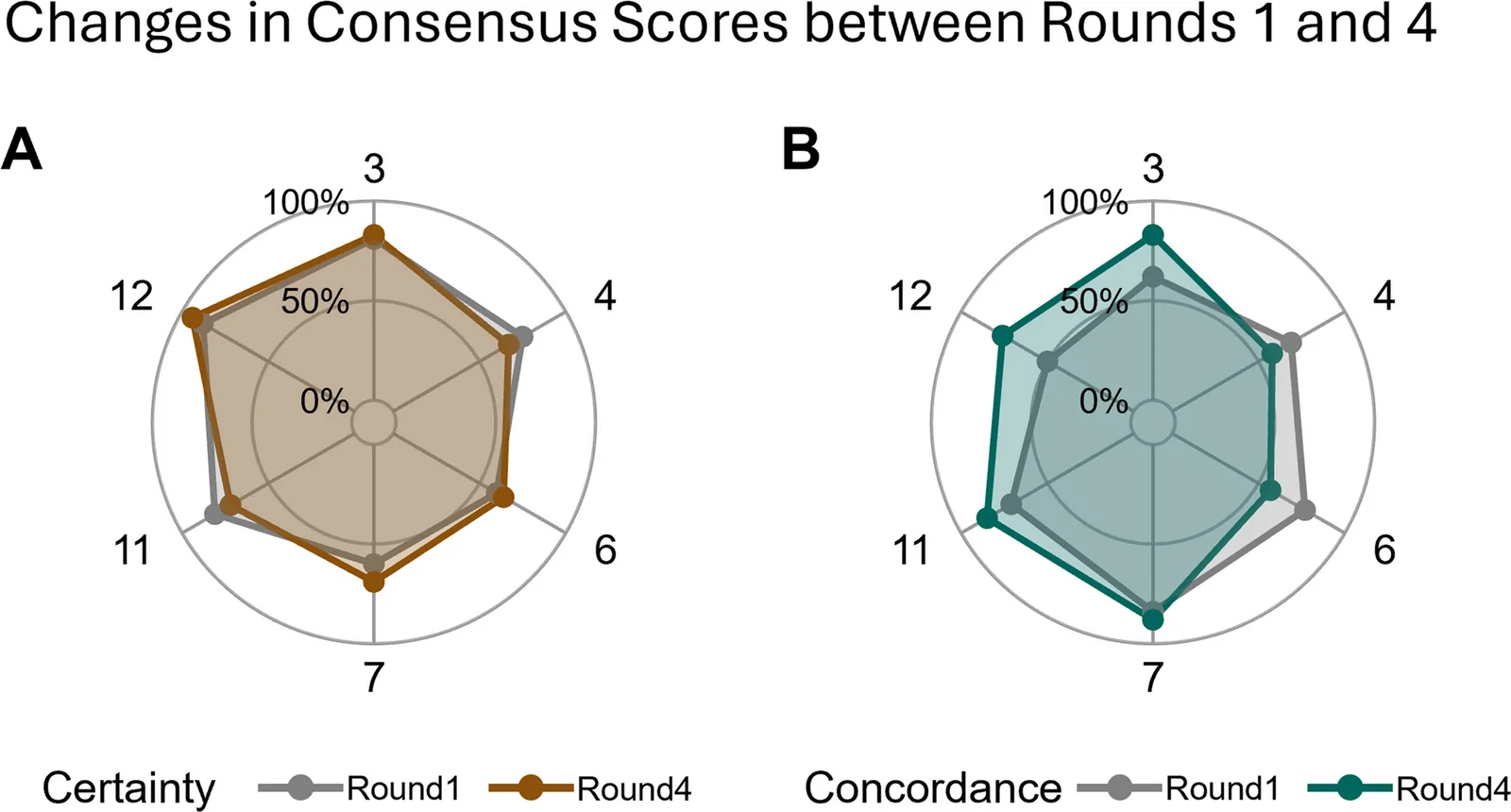
Spider plots showing changes in consensus scores between rounds 1 and 4. **A** Certainty and **B c**oncordance scores for each of the six statements are shown for round 1 (grey) and round 4 (brown and teal)

After the online discussion, the panel members were invited to reassess their Likert scale ratings of the six selected statements in the final Delphi round. Panel members who altered their ratings were asked to briefly explain the rationale for their change in opinion. The response rate in this round was 80%, resulting in 36 completed reassessment forms submitted within the timeframe. Figure 3 shows the change in certainty (Fig. 3A) and concordance (Fig. 3B) between *rounds 1* and *4*. Overall, changes in certainty were minimal, with the largest shift being a 9% increase for statement 7 and a 9% decrease for statement 11. Certainty increased for four of the statements after reassessment [3, 6, 7, 12] and decreased for two [4, 11].

Changes in concordance between *rounds 1* and *4* ranged from 4% (statement 7) to 26% (statement 12). Concordance increased for four statements [3, 7, 11, 12], while it decreased for statements 4 and 6. The largest change in concordance was observed for statement 12, which saw an increase of 26%. Nine of the ten panellists who changed their Likert scale rating for this statement shifted towards “Disagree”. The phrasing of statement 12: “A specific number of vessels should be revascularised Below-the-Knee (BTK) for optimal patient outcomes.”, was interpreted by many to mean “as many vessels as possible”. During the discussion, panel members agreed to take the literal interpretation of the statement, which led to a substantial increase in the concordance score after reassessment. Similarly, for statement 3, the wording “clearly defined ideal patient profile” was initially interpreted in different ways. While panel members noted that some lesion characteristics (e.g. calcification) present indications for atherectomy, they considered the phrasing “clearly defined” overly specific and consequently adjusted their Likert scale rating towards “Disagree”.

**Fig. 4:**
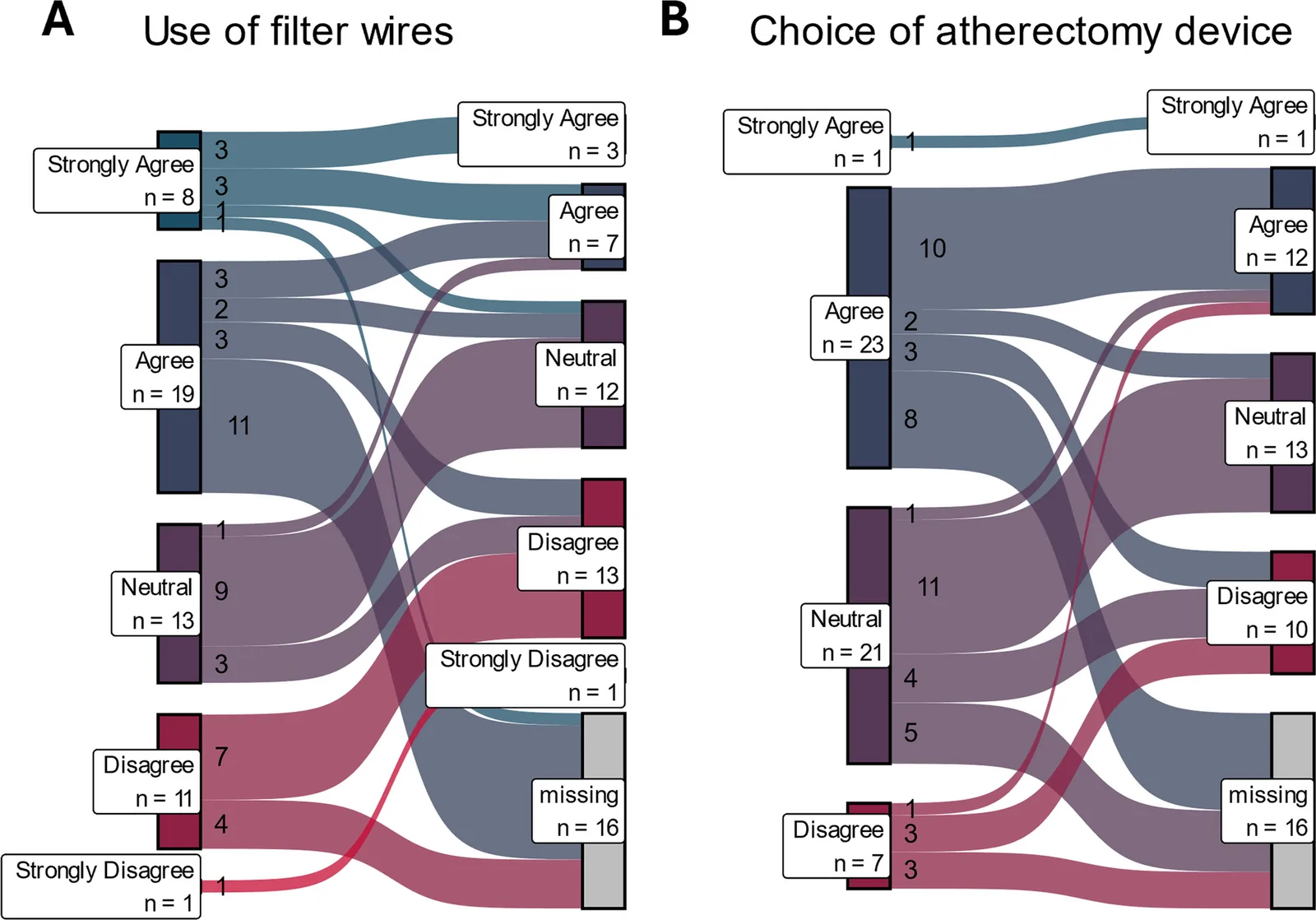
Sankey plots showing the drift in opinions between rounds 1 and 4 for two selected statements: **A** Statement 4 (The use of filter wires during atherectomy procedures is essential to prevent complications) and **B** Statements 6 (The choice of atherectomy devices significantly impacts patient outcomes). The left side of each graph shows Likert scale ratings from round 1 and the right side represents round 4. The flow of opinions is represented by the lines and can be followed from left to right. The thickness of the lines corresponds to the number of participants represented (small number)

For two statements, 4: “The use of filter wires during atherectomy procedures is essential to prevent complications.” and 6: “The choice of atherectomy devices significantly impacts patient outcomes.”), concordance decreased between rounds 1 and 4. Detailed changes in opinion are shown in Fig. 4. Thirty-six percent of participants changed their Likert scale rating for statement 4 and 30.6% of panel members changed their rating for statement 6. Negative change prevailed for both statements, with 12 out of 13 and 9 out of 11 panel members changing their rating towards “Disagree” for statements 4 and 6 respectively. A recurring theme in the discussion of both these statements was the fundamental heterogeneity among atherectomy devices, which operate through distinct mechanisms. Not all atherectomy devices currently on the market are compatible with filter wires, making generalised statements about their use difficult. Additionally, while panel members acknowledged that filters capture embolic debris, the clinical relevance of such debris was questioned, further swaying many opinions towards “Disagree”.

## Discussion

This Delphi consensus study aimed to identify topics where persistent disagreement or uncertainty points to research gaps in the treatment of peripheral artery disease by engaging a panel of experienced interventional radiologists. In that respect, the most important finding of the present study is that the Delphi process identified atherectomy as a particularly important subject for further investigation. While nine of the initial 17 statements reached consensus after the first round, statements addressing atherectomy did not converge in the same way and instead exposed areas where expert opinion remained divided despite structured feedback and reassessment.

Our consensus score combined two complementary measurements, each with a distinct interpretation. Low concordance scores reflect divergent expert opinions, while low certainty indicates limited confidence in the existing evidence base. Both metrics highlight areas with gaps in the current literature and guidelines.

The strongest signal emerged from statements 4 and 6, concerning the use of filter wires during atherectomy procedures and the impact of atherectomy device choice on patient outcomes. For both statements, concordance decreased between rounds 1 and 4, indicating that discussion did not resolve disagreement but rather sharpened awareness of the complexity of the issue. The significant heterogeneity among atherectomy devices and their compatibility with filter wires complicates general recommendations. The discussion round revealed that while filter wires efficiently capture embolic debris, the clinical relevance of such debris is not entirely clear. Additionally, increased complexity and cost of the procedure must be considered when making recommendations. While several studies have compared atherectomy devices, IVL and balloon-based techniques for vessel preparation in coronary arteries [12–16], the same evidence is not available for peripheral artery disease. A recent Cochrane review identified only seven studies (527 participants) comparing atherectomy with balloon angioplasty (BA) and atherectomy with BA and primary stenting, finding no clear superiority in terms of patency or major outcomes, but rated the certainty of evidence as very low [17]. Data on comparisons between different atherectomy technologies (orbital, rotational, directional or laser) is even sparser, with only a single prospective study having been published to date [18].

The landscape of clinical practice guidelines echoes this uncertainty. The Global Vascular Guidelines on the Management of Chronic Limb-Threatening Ischaemia date back to 2019 and emphasise individualised decision-making and the lack of high-level evidence for atherectomy compared to other modalities. While vessel preparation is acknowledged in the field as important for optimal outcomes, it remains poorly defined, with no standardised approach across device classes or lesion types. These findings emphasise the need for robust, comparative research and clearer guidelines on device selection and adjunctive measures.

Additionally, our findings also showed high agreement for several statements related to clinical practice organisation, including outpatient structures, ward rounds and follow-up pathways. This suggests that, among experienced interventional radiologists, there is strong consensus that procedural care in PAD should extend beyond the intervention itself.

This study does not exhaustively address the treatment algorithms for PAD. Included statements were selected by a small group of expert IRs and were not based on a systematic literature review but on perceived controversy, resulting in a selection that is biased by the interests and expertise of the group. Limitations of this study include the extended duration of the process which led to a relatively high dropout rate across rounds, and potential ambiguity in the phrasing of some of the initial statements. Especially for statement 12, clarification of the intended interpretation during the discussion round led to a strong increase in concordance after reassessment. This increase needs to be interpreted differently than similar changes for other statements, as it primarily reflects improved understanding rather than a true shift in expert opinion. These observations underscore that precise wording of initial statements is critical and will directly influence both the quality and the outcome of a Delphi study. We did not adapt the wording of any statement, to keep comparability of consensus scores across rounds as good as possible. However, adaptation of the wording should be considered, especially if reaching consensus among panel members is the explicit goal of the consensus study in question.

## Conclusion

This study demonstrates that the Delphi method is a valuable tool for identifying research priorities in PAD. Our findings point to critical gaps in evidence, particularly regarding atherectomy techniques and device comparisons, and call for the development of formalised, multidisciplinary guidelines to standardise clinical practice for vessel preparation. Critically, the lack of high-level evidence is a major bottleneck in the development of such guidelines. Randomised studies as well as high-quality observational studies on the use of atherectomy in diverse clinical scenarios are needed to advance this field and optimise care for patients suffering from PAD. While our study points to specific gaps in the literature, the heterogeneous presentation of PAD in patients makes the continued research in all areas of PAD management critical to ensuring the best possible care.

## Supplementary Information


Supplementary Material 1: Supplementary Table S1.Supplementary Material 2: Supplementary Table S2.

## Abbreviations

PAD: Peripheral artery disease
EBIR-ES: European Board of Interventional Radiologists—Endovascular Specialist
CLTI: Chronic limb threatening ischaemia
BTK: Below-the-knee
IVL: Intravascular lithotripsy.
BA: Balloon angioplasty
*n*: Number
