# Supplementary material for: Identifying research areas in peripheral artery disease treatment—a Delphi consensus approach
Source: CVIR Endovasc. 2026 Aug 27;9:109. doi: 10.1186/s42155-026-00762-z (PMC13522282; doi:10.1186/s42155-026-00762-z)
Supplement: Supplementary file 1 [file 42155_2026_762_MOESM1_ESM.docx]

**Supplementary Table 1**

| List of Delphi Panel Members | | |
| --- | --- | --- |
| Title | **First Name** | **Last Name** |
| Prof. | Nasreddin | Abolmaali |
| Dr. | Nasser | AlGharem |
| Dr. | **Omran** | **Almokdad** |
| Dr. | Arturo | Alvarez Luque |
| Dr. | **Tiago** | **Bilhim** |
| Prof. | **Christoph Andreas** | **Binkert** |
| Dr. | **Sanne** | **de Boer** |
| Prof. | **Michiel** | **de Haan** |
| Prof. | **Fabrizio** | **Fanelli** |
| Prof. | **Gerard** | **Goh** |
| Prof. | **Jan Peter** | **Goltz** |
| Prof. | **Patrick** | **Haage** |
| Prof. | **Mohamad** | **Hamady** |
| Dr. | **Jules** | **Harvey** |
| Dr. | **Can** | **Hazar** |
| Prof. | **Hanno** | **Hoppe** |
| Prof. | Rolf W. | Hügli |
| Prof. | Thomas | Jahnke |
| Dr. | **Elias** | **Kehagias** |
| Dr. | **Roman** | **Klöckner** |
| Dr. | **Michael** | **Kostrzewa** |
| Dr. | **Mateusz** | **Krasun** |
| Dr. | **Miltiadis** | **Krokidis** |
| Dr. | Aman | Kumar |
| Prof. | **Michael** | **Lee** |
| Dr. | **Igor** | **Litvin** |
| Dr. | **Romaric** | **Loffroy** |
| Dr. | **Jernej** | **Lučev** |
| Prof. | **Andreas H.** | **Mahnken** |
| Dr. | **Peter** | **Meid** |
| Prof. | **Robert A.** | **Morgan** |
| Prof. | **Stefan** | **Müller-Hülsbeck** |
| Dr. | **Arash** | **Najafi** |
| Dr. | Niklas | Nyman |
| Prof. | **Gerard** | **O'Sullivan** |
| Prof. | Rohit | Philip Thomas |
| Prof. | **Thomas** | **Rodt** |
| Dr. | Jaume | Sampere Moragues |
| Dr. | Christian | Scheurig-Münkler |
| Dr. | Jens | Schneider |
| Dr. | **Pankaj** | **Sharma** |
| Dr. | Jozef | Sivak |
| Prof. | Christof-Matthias | Sommer |
| Prof. | **Stavros** | **Spiliopoulos** |
| Dr. | Erik | Stahlberg |
| Dr. | **Jakob** | **Swanberg** |
| Dr. | Tobias | Trabold |
| Dr. | Grigorios | Tsoukalos |
| Dr. | **Christiaan** | **van der Leij** |
| Dr. | **Filipe** | **Veloso Gomes** |
| Prof. | **Christian** | **Wissgott** |
| Prof. | **Florian** | **Wolf** |

**Supplementary Table 1.** List of all Dephi panel members. The 36 panellists who participated in the final round of the Delphi consensus panel are highlighted in bold.
