# Supplementary material for: Identifying research areas in peripheral artery disease treatment—a Delphi consensus approach
Source: CVIR Endovasc. 2026 Aug 27;9:109. doi: 10.1186/s42155-026-00762-z (PMC13522282; doi:10.1186/s42155-026-00762-z)
Supplement: Supplementary file 2 [file 42155_2026_762_MOESM2_ESM.docx]

| Statement | Summary |
| --- | --- |
| **3. There is a clearly defined ideal patient profile for atherectomy procedures** | 31% of participants answered either Agree or Strongly Agree, they mainly cited heavily calcified lesions or recurrent stenosis as indications for atherectomy. Patients not being candidates for e.g. POBA or bypass or the failure of previous treatments was cited by others. 19% of answers were Neutral and 50% of participants answered Disagree or Strongly Disagree. The reasons given were mostly concerning a lack of clinical evidence to show the effectiveness of atherectomy, as well as a lack of SOPs and guidelines. It was also pointed out that many patients benefitting from atherectomy might similarly benefit from different techniques, making it an individual decision rather than a fixed patient profile. |
| **4. The use of filter wires during atherectomy procedures is essential to prevent complications** | 52% of participants either Agreed or Strongly Agreed with this statement. The reasons given were: filter wires reduce the risk of distal embolization as well as allow for more aggressive plaque removal, it was however pointed out that risk of embolization depends on atherectomy device used. This sentiment was also reflected by participants answering Neutral (25%), Disagree or Strongly Disagree (23% combined). Additionally, participants here pointed out that it makes the procedure more complicated and expensive. |
| **6. The choice of atherectomy devices significantly impacts patient outcomes** | 46% of participants chose either Agree or Strongly Agree, pointing out that different devices have varying mechanisms and capabilities that are optimized for different patient scenarios. However, operator experience with the atherectomy device was cited to be an important factor impacting success rate. This sentiment was reflected in the opinions of participants choosing Neutral (40%) and Disagree (14%). Furthermore, the lack of evidence comparing different devices with regards to patient outcomes was highlighted. |
| **7. IVL is a comparable alternative to atherectomy for specific patient scenarios** | 50% of participants either Agreed or Strongly Agreed with this statement. It was pointed out that both techniques are part of vessel preparation, and aim at heavily calcified lesions. They have, however, different modes of function, with IVL being able to crack open calcified lesions but not remove the calcium. These different modes of function were also cited by participants choosing Neutral (40%) or Disagree (10%), suggesting maybe a different interpretation of the wording “comparable alternative”. Additionally, a lack of evidence, guidelines, and SOPs was cited and the opinion stated that the use of either IVL or atherectomy was dependent on the patient case. |
| **11. There are clear indications for when to treat occluded stents versus opting for bypass surgery** | 22% either Agreed of Strongly Agreed with this statement. Reasons given included that it is commonly accepted that an endovascular approach should be tried first. Patient characteristics that indicate treatment of occluded stents were also listed (see below). 19% answered Neutral, citing mostly a lack of evidence and discordant SOPs, but also pointing out that the decision to treat with atherectomy vs bypass surgery was individual to the patient. Both these sentiments were reflected in the Disagree answers (56%). |
| **12. A specific number of vessels should be revascularized Below-the-Knee (BTK) for optimal patient outcomes** | 45% of participants chose Agree or Strongly Agree, but the answers given ranged from at least 1 vessel to 2-3 if possible. Overall, the sentiment for all given opinions, was that restoring flow to as many vessels as possible was the best option. Participants answering Neutral (12%), Disagree or Strongly Disagree (44%) additionally pointed out that the number of vessels that should be tried to be revascularized additionally depends on the existing collaterals and microcirculation, and that an angiosome approach is helpful in many cases. |

**Supplementary Table 2**
